# Apatinib combined with camrelizumab in the treatment of recurrent/metastatic nasopharyngeal carcinoma: a prospective multicenter phase II study

**DOI:** 10.3389/fimmu.2023.1298418

**Published:** 2024-01-03

**Authors:** Yunyan Mo, Yufei Pan, Bin Zhang, Jian Zhang, Yixin Su, Zhengchun Liu, Meiqing Luo, Guanjie Qin, Xiangyun Kong, Rongjun Zhang, Yu Pan, Yi Liang, Defeng Wang, Yuejia Wei, Hengwei Chen, Wei Jiang

**Affiliations:** ^1^ Department of Oncology, Affiliated Hospital of Guilin Medical University, Guilin, China; ^2^ Department of Radiation Oncology, Nanxishan Hospital of Guangxi Zhuang Autonomous Region, Guilin, China; ^3^ Department of Radiation Oncology, Wuzhou Red Cross Hospital, Wuzhou, China; ^4^ Department of Radiation Oncology, Laibin People’s Hospital, Laibin, China; ^5^ Department of Radiation Oncology, Lingshan County People’s Hospital, Qinzhou, China; ^6^ Department of Radiation Oncology, Affiliated Hospital of Guilin Medical University, Guilin, China; ^7^ Key Laboratory of Oncology (Guilin Medical University), Education Department of Guangxi Zhuang Autonomous Region, Guilin, China

**Keywords:** nasopharyngeal carcinoma, clinical trial, immunotherapy, targeted therapy, apatinib, camrelizumab

## Abstract

**Background:**

Preclinical studies demonstrated that immune checkpoint inhibitors combined with antiangiogenic drugs have a synergistic anti-tumor effect. This present phase II trial aimed to evaluate the efficacy and safety of apatinib combined with camrelizumab in patients with recurrent/metastatic nasopharyngeal carcinoma (RM-NPC).

**Methods:**

Patients with RM-NPC were administered with apatinib at 250 mg orally once every day and with camrelizumab at 200 mg *via* intravenous infusion every 2 weeks until the disease progressed or toxicity became unacceptable. The objective response rate (ORR) was the primary endpoint, assessed using RECIST version 1.1. Progression-free survival (PFS), overall survival (OS), disease control rate (DCR) and safety were the key secondary endpoints. This study was registered with ClinicalTrials.gov, NCT04350190.

**Results:**

This study enrolled 26 patients with RM-NPC between January 14, 2021 and September 15, 2021. At data cutoff (March 31, 2023), the median duration of follow-up was 16 months (ranging from 1 to 26 months). The ORR was 38.5% (10/26), the disease control rate (DCR) was 61.5% (16/26), and the median PFS was 6 months (IQR 3.0-20.0). The median OS was 14 months (IQR 6.0-21.25). Treatment-related grade 3 or 4 adverse events occurred in seven (26.9%) patients, and comprised anemia (7.7%), stomatitis (3.8%), headache (3.8%), pneumonia (7.7%), and myocarditis (3.8%). There were no serious treatment-related adverse events or treatment-related deaths.

**Conclusion:**

In patients with RM-NPC, apatinib plus camrelizumab showed promising antitumor activity and manageable toxicities.

## Introduction

Nasopharyngeal carcinoma (NPC) is a kind of head and neck cancer with special geographical distribution, with the highest incidence in South China, Southeast Asia and North Africa.(1) Although the 5-year overall survival rate of patients with NPC is around 80%, local recurrence and distant metastasis are still the leading causes of NPC treatment failure.(2, 3) Despite many clinical trials, consensus regarding the treatment of recurrent and/or metastatic nasopharyngeal carcinoma (RM-NPC) after first-line treatment has not been reached.

A fundamental feature of NPC is the association with Epstein-Barr virus (EBV) infection.(4) In addition, 89–95% of NPC express programmed death-ligand 1 (PD-L1), with more than 50% of malignant cells being PD-L1 positive in the majority of these tumors, which raised the possibility of immunotherapy for NPC.(5) A previous study (KEYNOTE-012) showed that the objective response rate (ORR) of pembrolizumab in recurrent or metastatic squamous cell carcinoma of the head and neck was 12%.(6) The ORR of nivolumab in RM-NPC was only 20.5%.(7) Even those patients with RM-NPC who were PD-LI-positive (KEYNOTE-028), the ORR of pembrolizumab was only 25.9%.(8) Therefore, the creation of novel combination treatments is urgently required to overcome PD-1 blockade resistance in advanced NPC. A previous study found that vascular endothelial growth factor (VEGF) was overexpressed in most (> 60%) clinical biopsy specimens of NPC.(9) Apatinib is a new tyrosine kinase inhibitor for VEGFR-2. Our previous study demonstrated the efficacy and safety of apatinib to treat RM-NPC.(10) Other studies confirmed that apatinib has efficacy in patients with RM-NPC, with a low incidence of severe toxicities.(11, 12) The above studies suggested that immunotherapy alone or targeted therapy alone is only moderately effective in RM-NPC. Preclinical studies showed that immunotherapy combined with anti-angiogenic drugs could improve the therapeutic effect.(13–15) However, the application of the apatinib plus camrelizumab in advanced NPC has rarely been reported.

Based on these results, we conducted an open-label, phase II study to evaluate the anti-tumor activity and safety of apatinib combined with camrelizumab in patients with RM-NPC.

## Methods

### Design of the study and its participants

This was a multicenter, open-label, single-arm, phase II clinical trial conducted at five hospitals in Guangxi, China, where the morbidity and mortality of NPC is high.(2, 16) The trial aimed to assess the antitumor efficacy and safety of apatinib combined with camrelizumab in patients with RM-NPC.

Inclusion criteria: (1) Male or female patients: 18–70 years old; (2) patients with NPC confirmed by pathology; (3) local recurrence and/or distant metastasis after one comprehensive treatment, and progression following at least first-line treatment; (4) Eastern Cooperative Oncology Group (ECOG) physical status score: 0–1; (5) at least one measurable lesion evaluated using Response Evaluation Criteria in Solid Tumor sversion1.1 (RECIST1.1); (6) expected survival ≥ 6 months; (7) the body’s major organs are functioning well; and (8) with satisfactory compliance and follow-up, the subjects willingly participated in the study and completed an informed consent form. The exclusion criteria were as follows: current autoimmune illnesses or a history of autoimmune diseases; diseases requiring immunosuppressive drugs, including congenital or acquired immunodeficiency diseases, active hepatitis B or C virus infection, active infection or uncontrollable heart disease; invaded important blood vessels by tumor; swallow disability; mental disorders; pregnancy; and prior anti-PD-L1 or anti-PD-1 antibody therapy.

### Procedures

This study enrolled 26 eligible patients with RM-NPC. The patients received 250 mg of apatinib orally once a day and camrelizumab 200 mg was injected intravenously once every two weeks until the disease progressed, intolerable toxicity appeared, the study was completed, or consent was withdrawn. Reduction of the dose of camrelizumab was not allowed. If the patient experienced a suspected immune related adverse event (AE), they could stop using camrelizumab temporarily or permanently, depending on the doctor’s judgment. Adjustments of the dose of apatinib were not permitted. Interruption of apatinib was allowed and was carried out according to the judgment of the therapist and local standard practice. If the dose of apatinib was interrupted, replenishment in subsequent cycles was not permitted, and if AEs greater than grade 3 occurred, treatment was discontinued.

Each investigator (WJ, YFP, JZ, BZ, and YXS) assessed the tumor response in accordance with RECIST version 1.1. All patients underwent baseline examinations, including laboratory tests, magnetic resonance imaging (MRI), or computed tomography (CT), bone scan (bone scintigraphy), within 28 days after study inclusion. Laboratory tests were carried out every two weeks, which included renal function tests, liver function tests, and standard complete blood counts. Every 4 weeks, an electrocardiogram, routine stool and urine tests, blood coagulation tests, and thyroid function tests were reexamined. Imaging examination was performed every 6 weeks until the disease progressed or follow-up treatment began. According to the National Cancer Institute Common Terminology Criteria for Adverse Events (version 4.03), we recorded AEs during the trial, during treatment, and within 3 months after cessation of treatment. At the end of the study, patient follow-up was carried out every 4 weeks by telephone until death or the end of follow up. Efficacy analysis was performed in the intention-to-treat (ITT) population. Safety analyses were performed on all patients who received at least one dose of study treatment.

Biomarker analyses employed blocks of paraffin-embedded tumor tissue taken from archival tumor tissue of the study participants. Immunohistochemical staining was performed after paraffin-embedded NPC tissue was cut into 5-µm-thick slides. The expression of PD-L1 was evaluated using a PD-L1 immunohistochemical kit (ab213524; Abcam, Cambridge, MA, USA) in the central laboratory. The comprehensive positive score (CPS) was used to report PD-L1 expression: CPS = (the number of PD-L1 positive tumor cells + the number of PD-L1 positive tumor associated immune cells)/the total number of tumor cells × 100. A CPS ≥ 1 indicated PD-L1 positivity. A primary antibody diluent was prepared using an antibody diluent (DAKO, Carpinteria, CA) containing background inducers and used for the following dilutions: anti-VEGFR-2 (ab115805, clone SP123,1:400; Abcam, Cambridge, UK); anti-CD4 (ab133616,1:800; Abcam); and anti-CD8 (ab245118,1:800; Abcam). Under light microscope, the whole section was randomly observed in 5 high power (10-40) visual fields, and the product of staining intensity and the percentage of positive cells was used as the judgment index. Finally, score of 0-7 was divided into low expression group, 8-12 was divided into high expression group. Real-time quantitative PCR was used to detect EBV DNA in plasma samples from patients. 400 copies/ml of plasma EBV DNA before treatment was taken as the critical value. A EBV DNA ≥ 400 copies/ml indicated EBV DNA positivity.

### Endpoints and assessment

The major endpoint was the objective response rate (ORR), which was determined by the investigators as the percentage of patients who had a complete response (CR) or a partial response (PR) according to RECIST version 1.1.

The secondary endpoints were: Progression-free survival (PFS), defined as the interval between treatment initiation and the date of the disease progression or death from any cause); overall survival (OS), defined as the interval between treatment initiation and the date of death from any cause; the disease control rate (DCR), defined as the percentage of patients with stable disease, PR, or CR; and safety.

### Sample size calculation

This clinical trial was a single-arm study with ORR as the primary endpoint. In accordance with Simon’s optimal two-stage design (one-sided α 5% and power 80%), the intended sample size was 26 patients. The response rate (RR) of nivolumab in RM-NPC was 20.5%.(7) Pembrolizumab (KEYNOTE-012) had an RR of 12% in cases of recurrent or metastatic head and neck squamous cell carcinoma. (6) Our trial included PD-L1-negative and PD-L1-positive patients; therefore, we estimated that the RR might be < 12% because the proportion of PD-L1 expression in patients was different. Considering the synergistic effect of apatinib and camrelizumab, we thought that the necessary condition for clinically significant anti-tumor activity in this confirmatory trial would be at least 27% additional ORR. Therefore, we set the ORR threshold as 12% and expected the ORR to be 39%. Seven patients were enrolled in the first stage. In the first stage, if one or more patients achieved an objective response (OR) (CR or PR), an additional 16 patients were included in the second stage. If not, the study would not progress to the next stage, and the trial would be stopped. The primary endpoint would be achieved if ≥ 5 of the 23 patients showed an OR in the second stage. We assumed that 10% of the patients would be lost to follow-up during the trial; therefore, the required sample size was 26 cases. Finally, the actual number of participants would prevail. The sample size was calculated using PASS (version 15) software.(17).

### Statistical analysis

All analyses were performed for all patients who were administered with at least one dose of the research treatment. The Clopper-Pearson method was used to determine the 95% confidence interval (CI) for ORR and DCR. PFS and OS were determined using the Kaplan-Meier technique. Statistics provided clinical and demographic features, as well as AEs. We used the χ2 test or Fisher’s exact test to evaluate the relationship between ORR and exploratory subsets, and the log-rank test was employed to compare the relationships among them. SPSS software (version 25) was used to carry out the statistical analyses. All statistical tests were bilateral tests, and *p* < 0.05 was considered statistically significant. The deadline for the analysis was March 31, 2023. This trial is registered at ClinicalTrials.gov, NCT04350190. Registered 16 April 2020, and enrolment is complete.

### Study oversight

All participating centers’ institutional review committees gave their approval for the trial program, which was carried out in compliance with the Declaration of Helsinki and the guidelines for good clinical practice. Before participating in the trial, each patient gave their informed consent in Chinese.

### Role of the funding source

The funders of the study had no role in study design, data collection, data analysis, data interpretation, as well as writing the report. The corresponding author had the sole responsibility for choosing to submit the report for publication, and had complete access to all the data generated during the study.

## Results

### Patient characteristics

**Figure 1:**
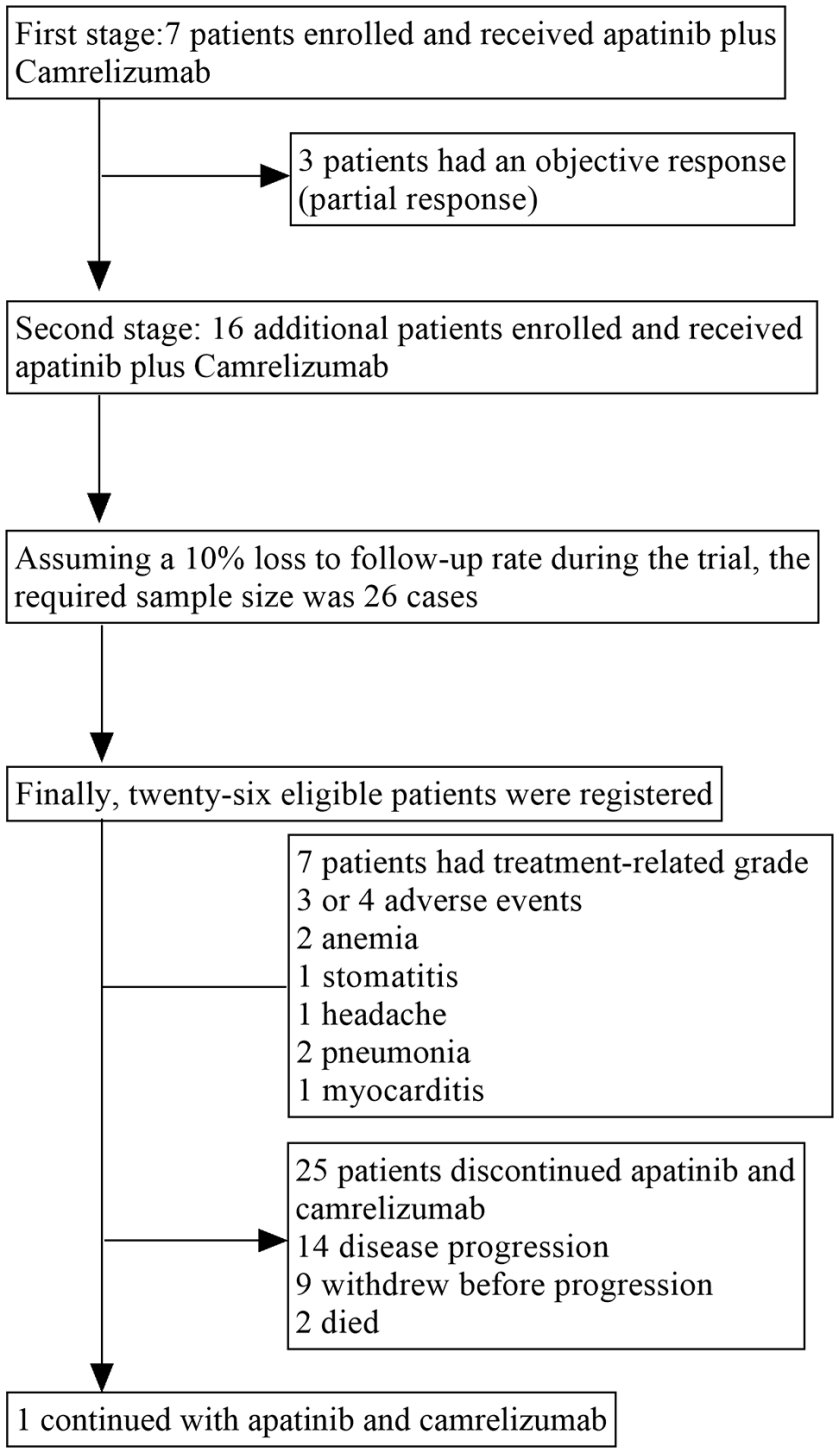
Trial profile.

In stage 1 of the trial, among the seven participating patients, three patients achieved an OR. Therefore, another 16 patients were recruited in the second phase. According to the assumed 10% loss to follow-up, finally, 26 eligible patients were registered between 14 January 2021 and 15 September 2021 ((ITT and safety set; **Figure 1**). There were 22 (84.6%) males and 4 (15.4%) females. Their ECOG performance status was 0 in 14 (53.8%) patients and 1 in 12 (46.2%) patients. All patients were pathologically classified as having undifferentiated non-keratinizing carcinoma. Their median age was 49 years old (range: 33–67). All (100%) patients had distant metastasis and one (3.8%) patient was associated with local recurrence. After disease progression on comprehensive treatment, and before being included in this study, 9 (34.6%) patients received first-line treatment, 9 (34.6%) received second-line treatment, and 8 (30.8%) received ≥ third-line treatment. Two patients discontinued study treatment before the first scheduled post-baseline scan: both had clinical deterioration deemed unrelated to the study regimen and died in 3 months. Therefore, treatment responses were evaluable only for 24 patients. Safety results of all 26 patients were analyzed. The data cut-off date was March 31, 2023. Follow-up was carried out for a median of 16 months (range: 1–26 months). At the data cutoff, 1 (3.8%) patient were undergoing treatment and 25 (96.2%) patients had discontinued the protocol treatment owing to disease progression (n=14), death(n=2), and withdrew before progression (n=9). **Table 1** summarizes the baseline characteristics of the participants.

**Table 1:** Baseline Characteristics of the patients (n = 26).

Characteristics	Patients
Age	
median (range)	49 (33-67)
Sex	
Male	22 (84.6%)
Female	4 (15.4%)
AJCC, 8th stage at initial diagnosis	
III	7 (26.9%)
IVa	8 (30.8%)
IVb	5 (19.2%)
unknown	6 (23.1%)
ECOG performance status	
0	14 (53.8%)
1	12 (46.2%)
**Histology**	
Squamous cell carcinoma	26 (100%)
EBV DNA	
≥400 copies/ml	9 (34.6%)
<400 copies/ml	13 (50%)
Unknown	4 (15.4%)
Recurrent or metastatic sites	
Nasopharynx	1 (3.8%)
Regional lymph nodes	2 (7.7%)
Lung	10 (38.5%)
Liver	10 (38.5%)
Bone	14 (53.8)
Number of previous lines of treatment before erolling in this study	
1	9 (34.6%)
2	9 (34.6%)
≥3	8 (30.8%)
PD-L1 expression status	
Positive	16 (61.5%)
Negative	5 (19.2%)
Unknown	5 (19.2%)
VEGFR-2 expression status	
High expression (score: 8-12)	8 (30.8%)
low expression (score: 0-7)	13 (50%)
Unknown	5 (19.2%)
CD4 expression status	
High expression (score: 8-12)	8 (30.8%)
low expression (score: 0-7)	13 (50%)
Unknown	5 (19.2%)
CD8 expression status	
High expression (score: 8-12)	2 (7.7%)
low expression (score: 0-7)	19 (73.1%)
Unknown	5 (19.2%)

Data are shown as the median (interquartile range (IQR)) or n (%). AJCC = American Joint Committee on Cancer. ECOG = Eastern Cooperative Oncology Group. EBV=Epstein–Barr virus. PD-L1= Program death-ligand 1. VEGFR-2=Vascular Endothelial Growth Factor Receptor-2.

### Antitumor activity

**Figure 2:**
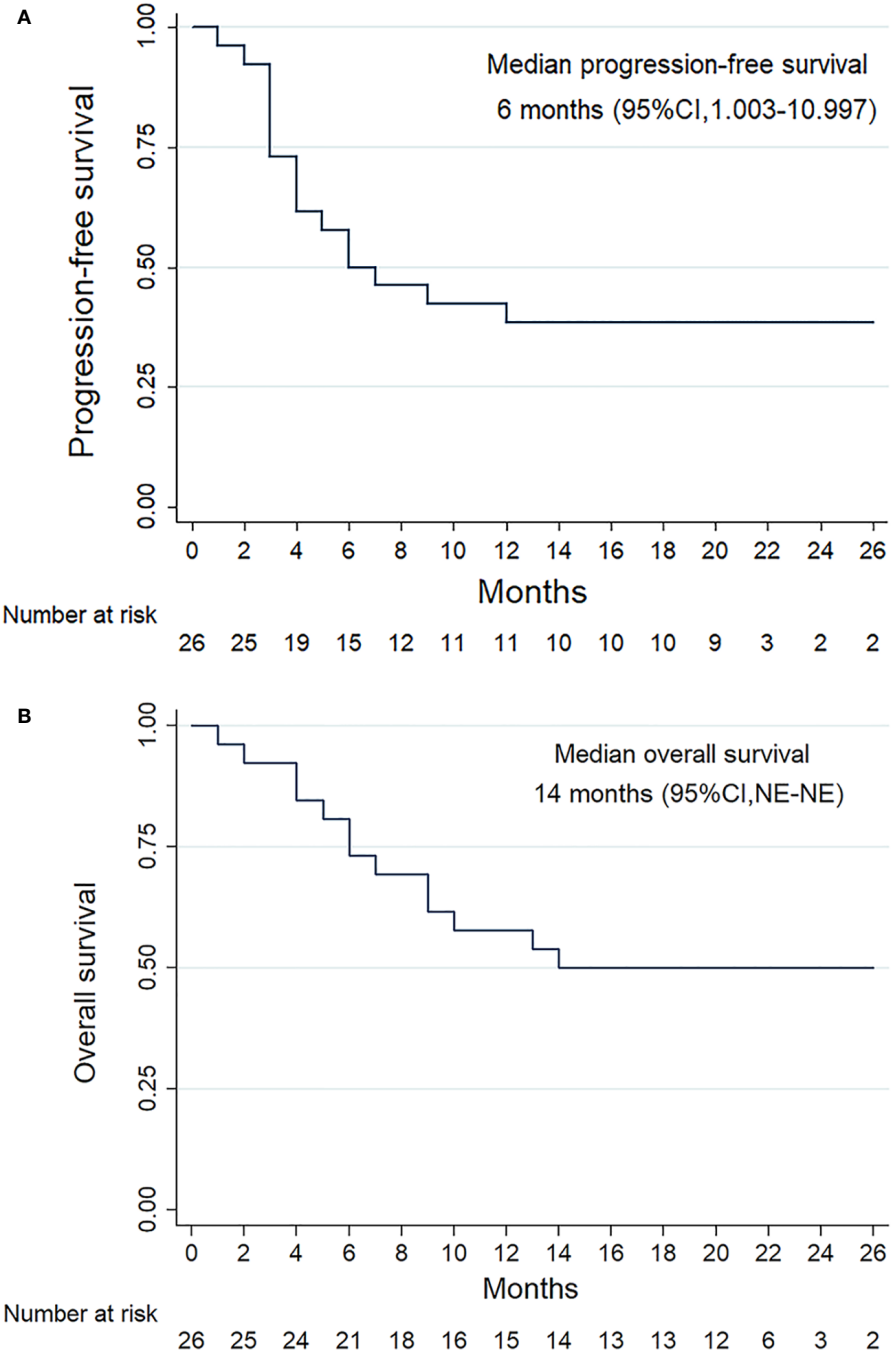
Survival outcomes of the patients with RM-NPC. Kaplan-Meier plots showing progression-free survival **(A)** and overall survival **(B)**.

**Figure 3:**
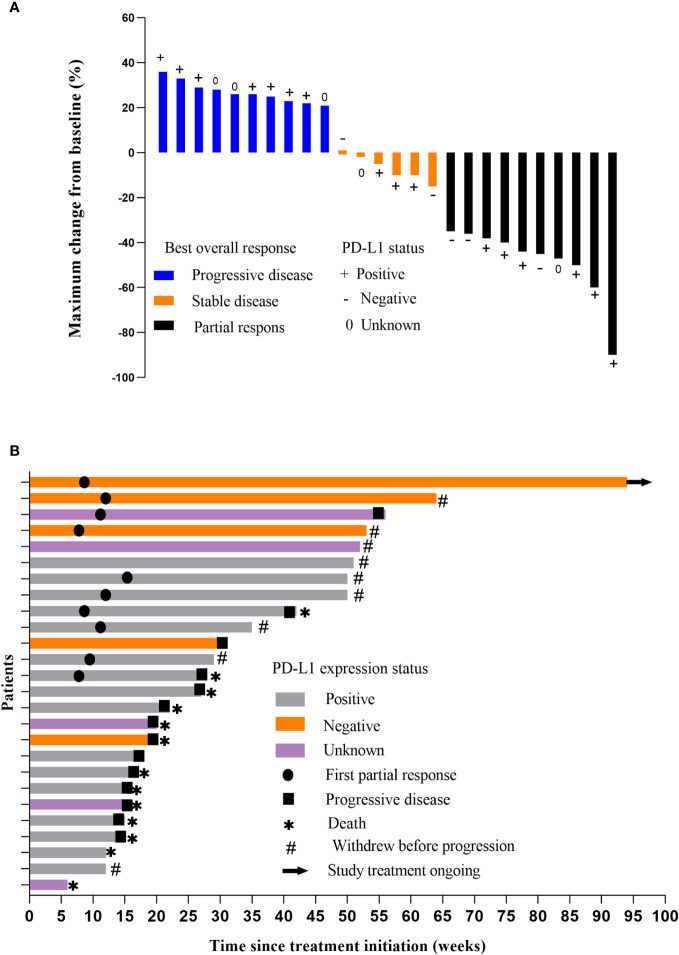
**(A)** Response of the tumors. Waterfall plot showing the maximum percentage change in tumor size compared with baseline for each patient. Measured according to Response Evaluation Criteria in Solid Tumors (version 1.1). **(B)** Time on treatment.

All patients were subjected to efficacy analysis. Fifteen patients (57.7%) in the efficacy evaluable population had smaller target lesions than those at baseline. Among these 15 patients, 10 achieved an OR according to RESIST1.1.(18) Among these 10 patients, all achieved a PR, but none achieved a CR. The ORR was 38.5% (10/26, 95% CI: 18.4– 58.5), and the DCR was 61.5% (16/26, 95% CI, 41.5–81.6) (**Table 2**). The median PFS(mPFS) was 6 months (IQR 3.0-20.0) (**Figure 2A**). The 1-year PFS rate was 46.2% (95% CI, 25.6–66.7). Thirteen patients died, resulting in median OS(mOS) being 14 months (IQR 6-21.25). (**Figure 2B**), the 1-year OS rate being 50% (95% CI, 29.4 -70.6). The tumor response is listed in **Table 2**, as shown in **Figure 3**.

**Table 2:** Tumor responses as assessed by the investigators or independent reviewers.

Efficacy variable	Number of patients [cases (%)]
Complete response	0 (0%)
Partial response	10 (38.5%)
Stable disease	6 (23.1%)
Progressive disease	8 (30.8%)
Objective response	10 (38.5%)
Disease control	16 (61.5%)
Not Evaluable^*^	2 (7.7%)

^*^Two patients discontinued study treatment before the first scheduled post-baseline scan.

### Safety

All 26 (100%) patients experienced treatment-related AEs of any grade. The most frequent AEs were anemia (57.7%), increased alkaline phosphatase (34.6%), and fatigue (30.8%) (**Table 3**). Anemia [in two (7.7%) patients], stomatitis [in one (3.8%) patient], headache [in one (3.8%) patient], pneumonia [in two (7.7%) patients], and myocarditis [in one (3.8%) patient] were among the grade 3 treatment-related AEs that occurred in 7 (26.9%) of the patients. Most of these events could be reversed by suspending apatinib and/or camrelizumab, or by administration of other drugs. There were no grade 4 treatment-related AEs or treatment-related fatalities. One of the patients who died had lung metastasis. Before death, the efficacy was evaluated as PR. The cause of death was poor control of local inflammation after nasopharyngeal radiotherapy, which damaged blood vessels and caused epistaxis, leading to death. The deaths of the rest of the patients (n = 12) were caused by progressive disease. Two (7.7%) patients discontinued apatinib as a result of treatment-related AEs (one with grade 3 stomatitis and one with persistent grade 2 decreased appetite). Three (11.5%) patients stopped using camrelizumab as a result of treatment-related AEs (two with grade 3 pneumonia and one with grade 3 myocarditis). The treatment-related AEs did not interrupt the study. **Table 3** shows all AEs, whether treatment-related or not.

**Table 3:** Treatment-Related Adverse Events in Total Treated Patients (n = 26).

Any treatment-related adverse event	Grade 1	Grade 2	Grade 3	Grade 4
Hypertension	5 (19.2%)	–	–	–
Anemia	8 (30.8%)	5 (19.2%)	2 (7.7%)	–
Proteinuria	4 (15.4%)	–	–	–
Increased AST	1 (3.8%)	–	–	–
Increased ALT	1 (3.8%)	–	–	–
Stomatitis	–	4 (15.4%)	1 (3.8%)	–
Fatigue	8 (30.8%)	–	–	–
Alkaline phosphatase increased	7 (26.9%)	2 (7.7%)	–	–
Neutropenia	2 (7.7%)	–	–	–
herpes zoster	1 (3.8%)	–	–	–
Hypothyroidism	3 (11.5%)			
Hyperthyroidism	–	–	–	–
Total bilirubin increased	–	1 (3.8%)	–	–
Reactive cutaneous capillary endothelial proliferation	3 (11.5%)	–	–	–
Fever	2 (7.7%)	1 (3.8%)	–	–
Headache	2 (7.7%)	1 (3.8%)	1 (3.8%)	–
Thrombocytopenia	1 (3.8%)	1 (3.8%)	–	–
Gingival pain	2 (7.7%)	–	–	–
Pruritus	2 (7.7%)	–	–	–
decreased appetite	5 (19.2%)	2 (7.7%)		
Myocarditis	–	–	1 (3.8%)	–
Pneumonitis	–	–	2 (7.7%)	–

### EBV DNA, PD-L1, VEGFR-2, CD4 and CD8 expression

Plasma EBV DNA was detectable before treatment in 22 of the 26 patients, but not in 4 patients. Of the 22 detectable patients, 9 were positive and 13 were negative. Their ORRs were 55.6% (5/9) and 38.5% (5/13), respectively, and there was no significant difference (P=0.666).

Among the 26 patients, 21(80.8%) had evaluable tumor biological samples to analyze the expression of PD-L1, VEGFR-2, CD4 and CD8 expression. Among these 21 patients, 16 (76.2%) had a PD-L1 CPS ≥ 1 and 14 (66.7%) patients had a PD-L1 CPS ≥ 10. In patients with a PD-L1 CPS ≥1, the ORR was 37.5% (6/16, 95% CI, 10.9–64.1) and the DCR was 56.3% (9/16, 95% CI, 28.9–83.6). The mPFS and mOS were 6 months and 13 months, respectively, in PD-L1-positive patients. PD-L1 negative patients had an mPFS of 14 months and mOS of 15 months. There is no significant difference in ORR was observed between patients with PD-L1-positive and PD-L1-negative tumors (37.5% vs 60.0%, P=0.611). In addition, patients with PD-L1-positive tumors had longer mPFS than patients with PD-L1-negative tumors.

Among the 21 patients, 8(38.1%) had high expression of VEGFR-2 and 13(61.9%) had low expression. In the patients with high expression of VEGFR-2, no patient reached the objective response. In the patients with low expression of VEGFR-2, 9(69.2%) patients reached the objective response. Among the 21 patients, 8(38.1%) had high expression of CD4 and 13(61.9%) had low expression. In the patients with high expression of CD4, 4(50.0%) patients reached the objective response. In the patients with low expression of CD4, 5(38.5%) patients reached the objective response.Among the 21 patients, 2(9.5%) had high expression of CD8 and 19(90.5%) had low expression. In the patients with high expression of CD8, 1(50.0%) patient reached the objective response. In the patients with low expression of CD8, 8(42.1%) patients reached the objective response.There was a significant difference in the ORR between VEGFR-2 high expression group and VEGFR-2 low expression group (0.0% vs. 69.2%, P =0.002). There is no significant difference in ORR was observed between patients with CD4 high expression group and CD4 low expression group (50.0% vs 38.5%, P=0.673). There is no significant difference in ORR was observed between patients with CD8 high expression group and CD8 low expression group (50.0% vs 42.1%, P=0.686).

## Discussion

In this multicenter, single-arm, phase II, prospective clinical trial, we reported the efficacy and safety of the VEGFR2-targeting apatinib combined with PD-1 inhibitor camrelizumab in patients with RM-NPC. Our findings revealed that the ORR of patients with RM-NPC treated with apatinib plus camrelizumab was 38.5% (10/26), the DCR was 61.5% (16/26), the mPFS was 6 months, and mOS was 14 months. Apatinib plus camrelizumab was well tolerated in patients with advanced NPC, regardless of their PD-L1 status, showing good ORR and PFS. Most treatment-related adverse events could be controlled by suspending apatinib and/or camrelizumab, or taking other drugs.

After receiving first-line chemotherapy, patients with RM-NPC who progress have few treatment alternatives. In the previous phase 1b KEYNOTE-028((NCT02054806) trial,(8) pembrolizumab treatment of those patients with RM-NPC who were PD-L1 positive resulted in 7 (25.9%) of the 27 PD-L1 positive patients with NPC achieving partial remission, 14 patients remained in a stable condition, but no patients showed complete remission. Another study of nivolumab in the treatment of PM-NPC, the international multicenter study of the Mayo clinical phase 2 consortium (NCI-9742), showed that ORR was 20.5%.(7) In a Phase II POLARIS-02 trial, toripalimab, a humanized IgG4 monoclonal antibody against PD-1, was used to treat patients with RM-NPC, resulting in an ORR of 20.5% and an mPFS of 1.9 months.(19) A study by Yang et al. on the treatment of RM-NPC with camrelizumab found that ORR was 28.2% and mPFS was 3.7 months.(20) Our study, which included both PD-L1-positive and PD-L1-negative patients, demonstrated that the ORR was significantly higher than that for immunotherapy alone, and the mPFS was prolonged.

Studies by Huang et al. found that the ORR of apatinib monotherapy in patients with RM-NPC was 31.4%, the mPFS was 3.9 months, and mOS was 5.8 months. (12)Li et al. found that the ORR of apatinib alone in the treatment of recurrent and refractory nasopharyngeal carcinoma was 31.37%.(11) Studies by Tao et al. found that the DCR of apatinib monotherapy for RM-NPC was 52.6% and the mPFS was 3.7 months.(21) Compared with the previous studies of apatinib in the treatment of advanced NPC, our study showed obvious advantages, such as prolonged mPFS, high ORR, high DCR, and low rate of severe adverse events, such as grade 3-4 hand-foot syndrome. In addition, in our study, we found that patients with low expression of VEGFR-2 had more objective responses than patients with high expression of VEGFR-2. We consider that the reason for this result is the small sample size and the selective bias of this study. Therefore, a further study with a larger sample size would be needed to explore the relationship between the efficacy of apatinib plus camrelizumab in patients with RM-NPC and the status of VEGFR-2 expression.

In our previous study (NCT03130270), apatinib at 500 mg/day was used as the initial dosage for all patients, as opposed to 250 mg/day in the current study.(10) Importantly, our present study showed that apatinib also has promising antitumor activity in RM-NPC with less toxicityies of treatment and higher treatment adherence of patients. In addition, in the present study, 73.1% of patients with lung and/or liver metastasis, which leading to poor prognosis, our trial still showed a good therapeutic efficacy. In summary, the appropriate dose of dual-drug (apatinib and camrelizumab) combination therapy has a good clinical application prospect.

Anti-angiogenic therapy can boost sensitivity to anti-PD-1/PD-L1 therapy by increasing PD-L1 expression and the infiltration of CD8+T cells into the tumor microenvironment, according to preclinical research.(15) In addition, related studies have shown that anti-angiogenic drugs can eliminate the immunosuppressive effect of the tumor microenvironment, which also suggests that the combination of immunotherapy and anti-angiogenic drugs might improve the therapeutic effect.(14) VEGFR-2 inhibitors play an immunomodulatory role, mainly by reducing regulatory T cells and myeloid suppressor cells, and promoting dendritic cell maturation and effector T cell infiltration.(13) Based on the above findings, it is believed that the combination of targeted antiangiogenic drugs and immune checkpoint inhibitors has the potential to treat a variety of cancers. In our study, the efficacy of anti-PD-1 antibodies plus VEGFR-2 inhibitors was superior that of immunotherapy alone in patients with advanced NPC.

Currently, scant investigations exist pertaining to the amalgamation of apatinib and camrelizumab in the therapeutic intervention of RM-NPC. A recent Phase II clinical study conducted by Ding et al. showed that the coadministration of apatinib and camrelizumab yielded an ORR of 65.5% and a DCR of 86.2% for RM-NPC.(22) Our present study uniqueness compared with the study by Ding et al. stems from the fact that the patient population differed between the studies. In the study by Ding et al., the majority of patients (82.8%) failed 1 line of treatment, and 17.2% failed ≥2 lines of treatment. However, the study population in our present study reflected a more treatment-resistant phenotype of patients, with 65.4% of patients having ≥2 lines of treatment. This could have influenced the differences between the results of studies testing the same regimen.

In this study, the observed adverse reactions were generally mild (23.6% grade 3–4 adverse events), and was similar to the results for apatinib combined with camrelizumab in other cancers.(23) Common toxicities, such as fatigue, anemia, and hypertension, can be controlled by dose reduction, dose adjustment, and supportive care. There were no patient deaths caused by adverse events. The most serious adverse reaction was pneumonitis, with an incidence of 7.7% (2/26). Fortunately, both cases of pneumonia in the patients improved after treatment, and both patients remain alive. Previous studies have shown that the incidence of reactive capillary hemangiomas in patients with NPC treated with camrelizumab alone was as high as 80%,(24) however, in our study, only 11.5% of patients were found to have reactive capillary hemangiomas, which might have been caused by apatinib resistance to camrelizumab. A study (NCI-9742) of Nivolumab alone in patients with RM-NPC found that 22% of patients had grade 3 or higher toxicities.(7) A phase II clinical trial (NCT02721589) of camrelizumab monotherapy in patients with RM-NPC showed that 16%(15/93) of the patients experienced grade 3 or 4 treatment-related toxicities.(24) The KEYNOTE-028 Study, comprising a study of pembrolizumab in patients with NPC, showed that 29.6% of patients had drug-related adverse events of grade 3 or more.(8) In studies of apatinib monotherapy in patients with NPC, Huang et al. found that 14.3% of patients had grade 3 adverse events,(12) and Li et al. found that 35.3% (18/51) of patients suffered grade 3–4 adverse events.(11) In our study, the combination of the two drugs increased the anti-tumor efficacy without significantly increasing the occurrence of adverse events. The combination of two drugs in the treatment of RM-NPC did not result in an increase in adverse events compared to monotherapy. We attribute this observation to several factors. First, the dose of apatinib used in the combination is halved compared to its single agent counterpart, thereby mitigating apatinib-induced adverse events. Second, apatinib and camrelizumab have different toxicities,(25, 26) therefore, their co-administration does not exacerbate toxicity. As a result, the safety profile of the combined regimen is manageable.

This study has a number of limitations. First, the small number of patients included made it less likely that the effectiveness observed was indeed successful, hence the results for anti-tumor activity were at the preliminary stage. Second, the patients had an ECOG performance status of 0-1 from southern China, which suggests that a selected population was enrolled in the study. Therefore, a further study would be needed to investigate the efficacy of this combination for non-Chiness patients with RM-NPC. Third, we excluded patients with tumor infiltration into major blood vessels from the study cohort to reduce the risk of bleeding during treatment. In subsequent studies, we will continue to focus on evaluating the post-treatment bleeding susceptibility of patients. Fourth, this was a single-arm study with no control group for comparison, and thus selection bias could not be ruled out.

## Conclusions

Our findings indicated that in patients with RM-NPC, camrelizumab plus apatinib exhibited promising antitumor efficacy and acceptable toxicity. These results support the view that anti-angiogenic drugs combined with immunosuppressants represent a new and promising anti-tumor combination regimen for RM-NPC. To confirm our findings, larger randomized controlled trials are required.

## Data availability statement

The original contributions presented in the study are included in the article/supplementary material. Further inquiries can be directed to the corresponding author.

## Ethics statement

The studies involving humans were approved by Affiliated Hospital of Guilin Medical University, Nanxishan Hospital of Guangxi Zhuang Autonomous Region, Wuzhou Red Cross Hospital, Laibin people’s Hospital, Lingshan County people’s Hospital. The studies were conducted in accordance with the local legislation and institutional requirements. The participants provided their written informed consent to participate in this study.

## Author contributions

YM: Writing – original draft, Writing – review & editing. YufP: Writing – original draft. BZ: Writing – original draft, Writing – review & editing. JZ: Writing – original draft, Writing – review & editing. YS: Writing – original draft, Writing – review & editing. ZL: Writing – original draft, Writing – review & editing. ML: Writing – review & editing. GQ: Writing – review & editing. XK: Writing – review & editing. RZ: Writing – review & editing. YuP: Writing – review & editing. YL: Writing – review & editing. DW: Writing – review & editing. YW: Writing – review & editing. HC: Writing – review & editing. WJ: Writing – original draft, Writing – review & editing.
